# Expired Patents

**Published:** 1906-06

**Authors:** 


### EXPIRED PATENTS.

January 22, 1906.

396,537, Rubber dam clamp, W. G. Browne, Atlanta, Ga.
396,566, Dental articulator, R. S. Hayes, East Bloomfield, New York.

February 5, 1906.

397,169, Dental engine, J. S. Campbell, London, England.
397,320, Process of making dental plates by electro-deposition, J. G. Ward, Irvington, N. J.

February 12, 1906.

397,556, Dental engine, J. H. Kidder, Lawrence, Mass.

The above list of patents is furnished by Davis & Davis, solicitors of American and foreign patents, Washington, D. C., and St. Paul building, New Pork City.

